# Child-adolescent emergency psychiatry: addressing false positive admissions

**DOI:** 10.3389/fpsyt.2024.1321702

**Published:** 2024-01-31

**Authors:** Linda Isaac, Tiphanie Sutton, Jasmine Kahlon, Pratima S. Pathania, Bradley L. Wolf, Ryan Pearce, Helen Iat Chio Chan, Matthew J. Zils

**Affiliations:** Kaiser Permanente Mental Health Training Program, Oakland, CA, United States

**Keywords:** emergency psychiatric services, child adolescent mental health, crisis, acute psychiatric admissions, pediatric

## Abstract

Current literature emphasizes the necessary and increasing role of the emergency department (ED) psychologist. This perspective paper will illustrate that the recent focus on an ED psychologist is necessary, but insufficient. Equally important, is an understanding of when a patient in a potential crisis does not require an ED admission, but rather an assessment that is made prior to the patient going to the ED. The essential role of an outpatient crisis team is vital in differentiating when an ED admission is indicated for a psychiatric crisis (true positive) and when an ED admission is not indicated for a psychiatric crisis (false positive). Evaluating crises prior to ED admissions accomplishes two critical healthcare objectives in a parallel process: 1) accurately assessing the proper level of care needed when a patient reports they are experiencing acute psychiatric symptoms (which may or may not necessitate emergency department level of care, and 2) reducing burden on an already over-extended ED when emergency care is not indicated. Our findings are uniquely drawn from a highly diverse youth patient population in Northern California, United States.

## Brief introduction

Emergency child psychiatry continues to be recognized as a growing subspeciality of psychology and psychiatry (1) with demands for skilled emergency department clinicians reaching an all-time peak during the COVID-19 pandemic (2) exposing a staggering number of youth in crisis. The extraordinary impact of COVID-19 on mental health has cast a spotlight on the specialty of psychiatry and the psychological cost of an unprecedented global lockdown has now been well-documented (3). The pandemic, while significant to understanding mental health, has exposed what many of us in emergency psychiatry were already well aware of: children and adolescents are at high risk for self-harm. Soaring case numbers of addiction (4), depression (5), and increased levels of intent to commit suicide (6) have all been highlighted in the literature. Of note is mental health concerns were among the highest for younger children and adolescents (7); a patient population that our team was tasked with treating while directly addressing the surge in demand for risk evaluations, hospital admissions and treatment interventions. Psychiatric pre-conditions have been exacerbated while countless individuals experienced their first onset of psychiatric symptoms during the pandemic (8) and those numbers are not relenting. Here we present evidence that specialized training in crisis assessment is necessary beyond those conducted in the context of an emergency department (ED), where patient presentation includes those with both clear and unclear suicidal (danger to self) or homicidal (danger to others) thoughts (or *ideations*). Perhaps more pressing is the decision-making that is necessary for the more ambiguous clinical presentations outside of the safe context of a hospital, in the absence of ongoing attentiveness of a dedicated medical team. The outpatient crisis specialists respond to referrals from multiple sources. We evaluate youth in crisis in the greater Oakland, CA area considered among the most diverse cities in the United States across multiple indexes: ethnicity, race, sexual orientation, gender identity, and socio-economic status. Thus, our patients represent a diverse patient population not readily accessible by other clinical and research teams. By this unique metric, our results are generalizable and informative to a readership with limited access to study this distinctive level of patient diversity.

## Crisis response team consult & liaison service

The crisis response team (CRT) is responsible for conducting risk assessments when patients are contemplating hurting themselves (danger to self) or someone else (danger to others), have engaged in past or present self-harm (cutting) or have symptoms of increasing instability and self-neglect (grave disability). Within an integrated healthcare System, CRT provides emergent and urgent care services that are critical to making the distinction between those patients who indeed need emergency level care (true positive) and those who present with acute psychiatric symptoms for which an emergency department admission is not indicated (false positive). CRT services include providing initial and ongoing consultation for Hospital medical floors (e.g., pediatric intensive care unit), addressing urgent mental health calls from other medical center clinics, outpatient clinic providers, and parents calling the clinic to report onset of acute psychiatric symptoms. The presenting problems are vast, complex, and multifaceted necessitating a CRT clinician who is decisive, resourceful, efficient and effective in utilizing established workflows to triage acute patients while maintaining a level of flexibility for a highly unpredictable clinical service line; in order to determine if a referral for medication evaluation, an outpatient individual therapy appointment, crisis stabilization services, an intensive outpatient program, emergency level care or a next day urgent outpatient evaluation is the most appropriate care. We suggest that in addition to the much-attended-to science of emergency department psychiatry, it is equally important to raise awareness of the crisis psychologist who can triage effectively *away* from the emergency department to reduce un-needed ED psychiatric visits to alleviate burden on an already strained emergency medical environment. Lead crisis psychologists (doctoral level clinicians) and senior crisis team members (Master’s Level clinicians) have extensive training and experience (> 8 years) in managing high risk patients. Standardized assessments (PHQ-9, Columbia Suicide Severity Rating Scale (C-SSRS) are utilized to support thorough risk consultations consisting of precise domains that must be collected by each crisis consultant (e.g. history of suicide attempts, self-injurious behaviors, substance use). The growing medical need of psychiatric patients in emergency rooms suggests that wider training of clinical providers is necessary as noted by Chou and Tseng (9) in the example of training emergency department nurses.Recommendations for proper level of care and decreasing avoidable ED admissions (false positives) are crucially important in ensuring a distressed patient is not needlessly waiting in an emergency department room, rather they are receiving clinically appropriate treatment to stabilize psychiatric symptoms and maintain safety. Efforts to reduce over-crowding in emergency rooms are paramount in the context of a medical center that is essentially tasked with triaging patients to appropriate care. Further burdening emergency departments is the wait time for higher risk patients to be transferred to an inpatient facility. From our experience, the wait time for pediatric patients to be placed at an inpatient facility can range from 24 hours to a week.

CRT clinicians must handle numerous clinical cases from multiple sources with a broad range of referral questions. Such case referrals can range from a Hospital Physician requesting a CRT consult for co-morbid medical and psychiatric symptoms (e.g. functional neurological disorder/Conversion Disorder), to a Pediatrician who refers a patient for an elevated PHQ-9 depression score, to an outpatient therapist who learns their patient has been contemplating suicide in the absence of a plan to end their life, or a parent who reaches out to the crisis line to report an oppositional teen who has engaged in cutting behavior. From the perspective of all the above noted referral sources, these are “crises,” but do they all require an admission to the emergency department? Thus, the crucial role for a CRT clinician is to triage patients effectively and efficiently – a skill that is essential to reducing unwarranted emergency department admissions, helping to mitigate the excessive burden on medical staff that was most pronounced during Covid-19 (see 10–12) and continues to serve as a default department for psychiatric emergencies. In a retrospective study by Shearer and Wang (13), the authors extracted pediatric emergency mental health admissions from the California Office of Statewide Health Planning and Development Dataset (2005-2015) and restricted the age range to patients between 5-17 years old. Among the 384,339 admissions reported by Shearer & Wang, “287,997 were discharged, 17,564 were admitted, and 78,725 were transferred.” These findings support the conclusion that emergency departments are increasingly serving as centers for urgent psychiatric stabilization. Consistent with our reporting, Shearer and Wang (13) conclude there is an insufficiency in the current system of care for managing pediatric patients who present with acute psychiatric symptoms.

The aim of this paper is to improve our understanding of the number of patients who do not need emergency department care, rather can be referred to a myriad of other non-emergency room treatment options serving the patient’s treatment needs and utilizing Hospital resources effectively.

## Treatment resources

### Medication consult

A patient being serviced by Child Psychiatry and Family Services Department’s Crisis Response Team (CRT) can be provided a medication consult in a variety of ways. Each day a Child Psychiatry and Family Services Department Licensed Psychiatrist is selected to be on-call and available for consultations for CRT. If the CRT team assesses a patient and deems the patient is in immediate need of a medication adjustment, evaluation, and/or consultation, CRT will place a referral with the on-call psychiatrist to meet with the patient and/or their legal guardian. These medication consults can take place in-person within the hospital ward floors, emergency department, and/or in the crisis walk-in clinic, or via telehealth consults via telephone or video visits. Then the psychiatrists conducting the consults will provide an update with the CRT team, as well as the patient’s care team (i.e., attending physician, PCP, individual psychiatrist).

### Outpatient services

When patients do not meet psychiatric HOLD criteria for inpatient level of care, patients are offered other services in the department. Depending on the patient’s and family’s needs, we offer outpatient services if patient does not already receive services from the Child Psychiatry department. The outpatient services include, individual therapy, group therapy, medication management, psychoeducational classes, and family therapy. If patients and families are interested in any of these services, we directly schedule them for appointments and do wrap around management by reaching out to the future providers/clinicians. The CRT team works closely with providers on the outpatient team and many of the outpatient providers have rotational experience in CRT as part of the yearly requirement in the Child Psychiatry Department. All outpatient providers are trained in crisis work and consult with CRT as needed for more complex patient risk presentations.

### Sub-inpatient acute & specialty psychiatric care

Within the Child & Family Mental Health Care in the East Bay Region, there are several levels of Psychiatric Service available to patients who do not meet Criteria for Inpatient Psychiatric Hospitalization.

### Crisis residential program

Patients may be referred to a Crisis Residential Program, consisting of residential placement for 14-30 day treatment on a contracted Specialty Mental Health Campus with full-time, 24-hour staffing and on-site Psychiatric management. Such Residential Programs are voluntary and must be agreed to by both the parent(s) and the patient. These programs often target specific diagnostic profiles (Severe Depression) and may include dual diagnosis treatment to address substance abuse concerns as well.

### Partial hospitalization program

Patients may be referred to a Partial Hospitalization Program, sometimes referred to as Day Treatment Program, run by contracted Acute Care Mental Health organizations in which the patient remains living at home with their parents/family while attending daily intensive Psychiatric Treatment for 4-6 hours per day Monday-Friday for 10 business days (weekends are not included). Such programs may offer specialty services to help stabilize acute symptoms of Depression, Panic, or OCD.

### Addiction medicine & recovery services

Also known as Chemical Dependency & Recovery Program (CDRP), this Substance Abuse treatment specialty is available to teens and adults on a voluntary basis and is considered an adjunct but separate Mental Health Specialty Department apart from Child & Adult Psychiatry and offers treatment targeting chemical substance abuse (this program does not treat behavioral addiction such as sexual addiction, gambling, or video gaming). Referrals to this program are appropriate for patients whose substance abuse patterns/frequency pose an obstacle to effective outpatient Psychiatric treatment.

### Crisis stabilization outpatient program

Run through the Outpatient Department of Child & Family Psychiatry, the Crisis Stabilization program is a temporary addition to regular Outpatient treatment with the patient’s primary therapist and consists of patient assignment to an outpatient therapist who specializes in reducing acute Psychiatric symptoms of self-harm and suicidal ideation over the course of 6-10 weeks. This program may also include referral to specialized group psychotherapy targeting acute symptoms utilizing psychoeducation, safety planning, and coping skills didactic work.

### Parity ongoing outpatient program

Run through the Outpatient Department of Child & Family Psychiatry, the Parity Program is for patients with chronic, complex diagnostic profiles who have severe symptoms that impact their functioning across multiple domains (home, school, social functioning) and for whom previous attempts at regular private practice or Outpatient Psychiatry treatment have been ineffective. This program may also include referral to specialized group psychotherapy and parent psychoeducation or coaching and is staffed by senior clinicians who specialize in chronic, complex patient presentations.

### Intensive outpatient program

The Intensive Outpatient Program (IOP) treats patients who have been evaluated to need a higher level of care while still in an outpatient setting that meets multiple times a week (3 days weekly) and for an extended time (2-3 hours each day). Crisis response clinicians can refer to the IOP to circumvent inpatient hospitalization or oftentimes an IOP referral will be made when a patient has completed inpatient hospital treatment. Symptom acuity is a key criterion for IOP. Specifically, there must be a clinical need for this higher level of care based upon a patient’s report of danger to self or others, or concerns about the patient’s ability to attend to their daily functioning needs. Once admitted to IOP, patients receive comprehensive care from psychotherapy to medication consultation.

## Methods

A patient tracking system was implemented in the crisis service line. Over a duration of 1.5 years from October 2020 – April 2022, the tracking system captured information on the referral source (e.g., emergency department, pediatric medical ward), presenting risk concern (e.g., danger to self) and final disposition and treatment plan (e.g., inpatient hospitalization, intensive outpatient treatment). We then calculated descriptive statistics to address our primary question of interest on how many patients were sent to the emergency department who did not require urgent psychiatric attention.

### Research questions

We put forth the following specific research questions:

1. How many psychiatric crisis referrals needed ED level of care (true positives)?
2. How many psychiatric crisis referrals did not need ED level of care (false positives)?

## Results

### Patients admitted to the ED prior to an outpatient crisis psychologist evaluation

In our early findings, we show that up to 40% of ED-admitted patients did not require emergency level care (false positives). Notably, this group of patients were already admitted to the ED prior to being evaluated by a crisis psychologist. See **Table 1**.

**Table 1:** Total Number of Patients in the ER (N= 120).

ED Patients (N=76) Disposition Numbers				
IP = 48	CS = 3	IOP = 1	MED = 4	OP = 20

True Positives = 63% (76 patients), False Positives = 37% (44 patients).

True positives = patients who did need ED level of care.

False positives = patients who did not need ED level of care.

### Patients admitted to the ED after an outpatient crisis psychologist evaluation

Next, we examined the false positive rate of patients who were evaluated by a crisis psychologist prior to an ED admission. Consistent with our argument, only 13% of these patients deemed to be high risk were dispositioned to the ED after a crisis psychologist conducted a risk evaluation in the outpatient setting. In other words, 86% of patients who would have presumably gone directly to the ED, did not need an emergency admission (false positives = 87%). See **Table 2**.

**Table 2:** Total Number of Patients Evaluated Prior to the ED (N = 118).

ED = 15	CS = 22	IOP = 2	MED = 15	OP = 64

True Positives = 13% (15 Patients), False Positives (103 Patients) = 87%.

True positives = patients who did need ED level of care.

False positives = patients who did not need ED level of care.

### Crisis triage calls

**Figure 1:**
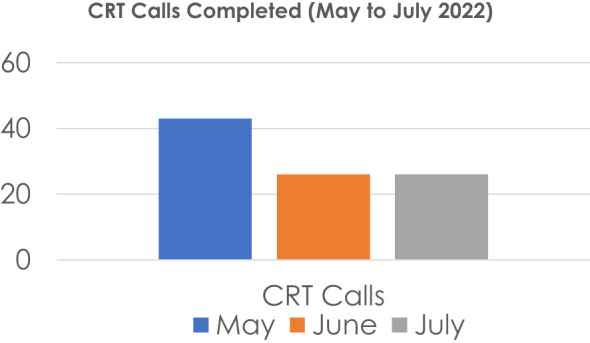
Total Number of Crisis Calls over 3 Months. Total Calls = 95, Presenting Concerns: Passive SI, Self-Harm, recent ED or inpatient hospitalization, ‘Unclear’, Cases Requiring 2+ calls: 19 (20%).

The crisis response teams are also responsible for triaging incoming crisis calls from various sources that largely includes parents calling the clinic and requesting to speak with a crisis clinician. We began tracking CRT calls and present the data below for calls between July-August 2022. See **Figure 1**.

Crisis calls represented a total of 61% of CRT encounters in the three-month duration. CRT triaging is essential to expediting patient care. Moreover, it is highly vital to the operational needs of a large department with high volume of patients. Triaging high risk patients with a telephone screen is essential in the face of the ongoing high demand for child adolescent psychiatry services. It is essential to avoid delays in proper treatment dispositioning with real-time crisis assessments.

## Discussion & conclusions

**Figure 2:**
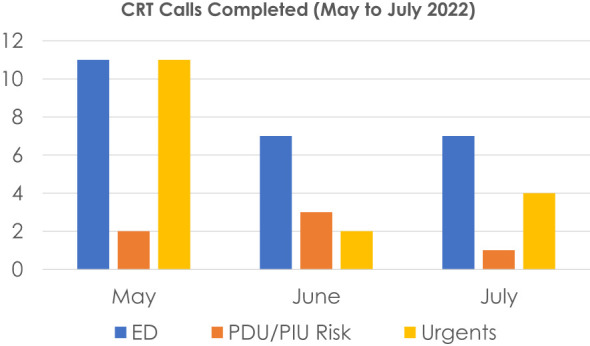
Number of Risk Consults Across 3 Months. ED, Emergency Department; PDU/PIU, Pediatric Medical Wards; Urgent: outpatient. Highest demand = May end of school year, Risk consults 90% Non-risk consult = 10%.

An accurate estimation of patients needing emergent care for a psychiatric crisis, and subsequently evaluating the possibly unnecessary burden on emergency departments, served as the impetus for this work. Our brief and simple descriptive statistics have successfully demonstrated that not all patients presenting with acute psychiatric symptoms should be deemed “in crisis,” nor should they immediately be dispositioned to an emergency department. We have detailed the multifaceted alternate psychiatric interventions that directly speak to our manuscript goals of patients receiving more clinically appropriate care via referrals for active treatments such as IOP, crisis stabilization, and PHP services; and simultaneously, minimizing the rate of false positives also defined here as patients who do not meet criteria for emergency level of care. Indeed, it is critical for clinicians to refer patients to the ER when they are at imminent risk for safety concerns such as danger to self or others (DTS/DTO) or gravely disabled (GD). While the COVID-19 pandemic encompassed a surge in child-adolescent ER psychiatric admissions, the lifting of Covid restrictions by no means has lifted the demand for more acute psychiatry services (14). We posit that an ER-dedicated psychologist is necessary but not enough to address the concern for patient false positives. A well-trained and specialized clinician dedicated to evaluating psychiatric crises and imminent risk factors *prior* to the ER is a necessary role if hospital systems are committed to both the proper level of care for psychiatric patients and alleviating the unnecessary burden on emergency department medical teams. See **Figure 2**.

## Data availability statement

The raw data supporting the conclusions of this article will be made available by the authors, without undue reservation.

## Ethics statement

Ethical approval was not required for the study involving humans in accordance with the local legislation and institutional requirements. Written informed consent to participate in this study was not required from the participants or the participants’ legal guardians/next of kin in accordance with the national legislation and the institutional requirements. This is a retrospective chart review only study with very minimal existing data. No demographics, no identifiable information, no details regarding diagnosis or any other identifiable marker was included. All clinical or subject information was excluded for maximum subject protection. Data was highly restricted. Research was conducted according to the principles of the Declaration of Helsinki. 

## Author contributions

LI: Conceptualization, Data curation, Investigation, Methodology, Project administration, Supervision, Validation, Writing – original draft, Writing – review & editing. TS: Data curation, Formal analysis, Writing – review & editing. JK: Data curation, Writing – review & editing. PP: Data curation, Writing – review & editing. BW: Data curation, Writing – review & editing. RP: Data curation, Writing – review & editing. HC: Data curation, Writing – review & editing. MZ: Data curation, Funding acquisition, Project administration, Resources, Writing – original draft, Writing – review & editing.
